# Supplementary material for: Structure-function analysis of the lithium-ion selectivity of the voltage-gated sodium channel
Source: J Gen Physiol. 2026 Feb 19;158(3):e202513855. doi: 10.1085/jgp.202513855 (PMC12919392; doi:10.1085/jgp.202513855)
Supplement: Table S1 — shows data collection and refinement statistics. [file jgp_202513855_tables1.docx]

**Supplementary Information**

**Structure-function analysis of the lithium-ion selectivity of the voltage-gated sodium channel**

Yuki K. Maeda^1,2^, Kentaro Kojima^2^, Tomoe Y. Nakamura^1^, Toru Nakatsu^2^, Katsumasa Irie^2^

1: Department of Pharmacology, Faculty of Medicine, Wakayama Medical University, Wakayama, Japan

2: Department of Biophysical Chemistry, Faculty of Pharmaceutical Sciences, Wakayama Medical University, Wakayama, Japan

*Address correspondence to: Katsumasa Irie, Department of Biophysical Chemistry, School of Pharmaceutical Sciences, Wakayama Medical University, 25-1, Shichibancho, Wakayama, 640-8156, Japan

Tel.: +81-73-488-1837; Fax: +81-73-488-1946; E-mail: [kirie@wakayama-med.ac.jp](mailto:kirie@wakayama-med.ac.jp)

**Supplementary Table 1. Data collection and refinement statistics.**

|  | **S178T^NK^** | **S178A^NK^** | **S178G^NK^ (LivAb^NK^)** | **S178T^NK/TA^** | **S178A^NK/TA^** |
| --- | --- | --- | --- | --- | --- |
| **Wavelength(Å)** | 1.00 | | | | |
| **PDB entry** | 9UC1 | 9UC2 | 9UC3 | 9UC4 | - |
| **Resolution range** | 45 - 2.6  (2.7 - 2.6) | 45 - 2.9  (3.0 - 2.9) | 44 - 3.0  (3.1 - 3.0) | 45 - 3.4  (3.5 - 3.4) | 45 - 4.1  (4.2 - 4.1) |
| **Space group** | *I* 4 2 2 | | | | |
| **Unit cell** | 128.0  128.0  200.3  90  90  90 | 126.6  126.6  201.5  90  90  90 | 128.1  128.1  200.4  90  90  90 | 128.7  128.7  202.0  90  90  90 | 127.4  127.4  203.2  90  90  90 |
| **Total reflections** | 377716  (32294) | 1921436  (173268) | 1186605  (111060) | 302043  (31081) | 511269  (33308) |
| **Unique reflections** | 25937  (938) | 18554  (824) | 17093  (748) | 12030  (527) | 6866  (381) |
| **Multiplicity** | 14.6  (12.8) | 103.6  (95.1) | 69.4  (67.0) | 25.1  (26.9) | 74.5  (50.0) |
| **Completeness (%)** | 86.69  (37.08) | 90.19  (45.23) | 90.68  (45.11) | 92.10  (45.51) | 94.03  (57.12) |
| **Mean I/sigma(I)** | 27.90  (1.32) | 24.02  (1.55) | 21.30  (1.20) | 10.66  (1.41) | 19.85  (2.66) |
| ***R*_merge_** | 0.051  (1.863) | 0.343  (5.400) | 0.160  (6.727) | 0.677  (7.837) | 0.349  (2.766) |
| ***R*_pim_** | 0.014  (0.537) | 0.037  (0.555) | 0.022  (0.823) | 0.142  (1.534) | 0.039  (0.392) |
| **CC1/2** | 1  (0.765) | 0.997  (0.832) | 0.997  (0.806) | 0.996  (0.743) | 1  (0.864) |
| ***R*_work_** | 0.259  (0.262) | 0.248  (0.296) | 0.273  (0.372) | 0.279  (0.300) | - |
| ***R*_free_** | 0.274  (0.296) | 0.282  (0.307) | 0.300  (0.467) | 0.299  (0.363) | - |
| **Number of non-hydrogen atoms** | 2345 | 2345 | 2276 | 2343 | - |
| **macromolecules** | 1871 | 1869 | 1804 | 1869 | - |
| **ligands** | 449 | 449 | 449 | 449 | - |
| **solvent** | 25 | 27 | 23 | 25 | - |
| **Protein residues** | 229 | 229 | 220 | 229 | - |
| **RMS(bonds)** | 0.004 | 0.014 | 0.013 | 0.004 | - |
| **RMS(angles)** | 0.82 | 1.77 | 1.54 | 0.74 | - |
| **Ramachandran favored (%)** | 98.24 | 98.24 | 98.15 | 99.12 | - |
| **Ramachandran allowed (%)** | 1.76 | 1.76 | 1.85 | 0.88 | - |
| **Ramachandran outliers (%)** | 0.00 | 0.00 | 0.00 | 0.00 | - |
| **Rotamer outliers (%)** | 0.00 | 0.00 | 0.00 | 0.00 | - |
| **Clash score** | 15.73 | 17.35 | 14.48 | 11.30 | - |
| **Average B-factor** | 91.18 | 80.37 | 91.37 | 112.20 | - |
| **macromolecules** | 88.64 | 78.44 | 89.96 | 110.18 | - |
| **ligands** | 103.54 | 90.45 | 99.29 | 122.13 | - |
| **solvent** | 59.47 | 46.79 | 47.04 | 85.41 | - |
